# Stem cell-based therapy for pulmonary fibrosis

**DOI:** 10.1186/s13287-022-03181-8

**Published:** 2022-10-04

**Authors:** Wenzhao Cheng, Yiming Zeng, Dachun Wang

**Affiliations:** 1 Stem Cell Laboratory, Second Affiliated Hospital of Fujian Medical University, Quanzhou, Fujian China; 2 The Brown Foundation Institute of Molecular Medicine for the Prevention of Human Diseases, University of Texas Medical School at Houston, Houston, TX USA

**Keywords:** Stem cells, Cell therapy, Extracellular vesicles, Exosomes, Pulmonary fibrosis

## Abstract

Pulmonary fibrosis (PF) is a chronic and relentlessly progressive interstitial lung disease in which the accumulation of fibroblasts and extracellular matrix (ECM) induces the destruction of normal alveolar structures, ultimately leading to respiratory failure. Patients with advanced PF are unable to perform physical labor and often have concomitant cough and dyspnea, which markedly impair their quality of life. However, there is a paucity of available pharmacological therapies, and to date, lung transplantation remains the only possible treatment for patients suffering from end-stage PF; moreover, the complexity of transplantation surgery and the paucity of donors greatly restrict the application of this treatment. Therefore, there is a pressing need for alternative therapeutic strategies for this complex disease. Due to their capacity for pluripotency and paracrine actions, stem cells are promising therapeutic agents for the treatment of interstitial lung disease, and an extensive body of literature supports the therapeutic efficacy of stem cells in lung fibrosis. Although stem cell transplantation may play an important role in the treatment of PF, some key issues, such as safety and therapeutic efficacy, remain to be resolved. In this review, we summarize recent preclinical and clinical studies on the stem cell-mediated regeneration of fibrotic lungs and present an analysis of concerning issues related to stem cell therapy to guide therapeutic development for this complex disease.

## Introduction

Pulmonary fibrosis (PF) is a type of interstitial lung disease in which the normal lung structure is replaced by a disrupted alveolar architecture containing aggregated proliferative fibroblasts and myofibroblasts in the extracellular matrix (ECM) [1]. There are several risk factors and fibrogenic triggers that initiate lung tissue damage and PF, such as smoking, virus infection, radiation, autoimmune reactions, aging, genetic elements and environmental exposure (e.g., asbestos and silica) [2]. There are two types of PF: those with known causes, such as sarcoidosis, pneumoconiosis and chronic hypersensitivity pneumonitis, and those with unknown causes, termed idiopathic PF (IPF), which represents the most common form of lung fibrosis in humans [1, 3]. IPF is an age-related, inversible interstitial lung fibrotic disease that is more prevalent in patients 50 years or older, with an incidence of 10–20 per 100,000 individuals in the United States and Europe [1]. Unfortunately, IPF patients have a poor prognosis, with a median survival of 2–4 years after diagnosis, and the mortality rate exceeds that of some malignancies [1, 2]. To date, lung transplantation is the only feasible curative therapy for patients with advanced PF.

Despite decades of study, the pathogenesis of PF is still unclear. The lung is a complex organ where O_2_/CO_2_ exchange occurs, and it is constantly exposed to many injurious entities that can destroy alveolar epithelial cells [3]. Interestingly, the lungs can repair or restore injured alveolar cells through a cascade of finely synchronized biological processes [4]. Alveolar epithelial cells consist of a combination of alveolar type I (ATI) and alveolar type II (ATII) cells. ATI cells are large squamous cells that cover more than 90% of the alveolar surface area; these cells function as the epithelial component of the air-blood barrier, connect to pulmonary capillaries, and provide an interface for gas exchange. ATII cells have critical secretory, metabolic and immunological functions and are progenitor cells for alveolar epithelial cells that can self-renew and differentiate into ATI cells and that maintain the integrity of the alveolar epithelium. Accumulating evidence indicates that ATII injury is an early event in the onset of PF [5]. Repetitive injury to the alveolar epithelium can cause stochastic profibrotic epigenetic reprogramming, premature and persistent epithelial cell senescence, and the secretion of multiple mediators, including transforming growth factor-β (TGF-β), fibroblast growth factors (FGFs), cytokines, and coagulants, that induce the activation and proliferation of fibroblasts and myofibroblasts [3]. Indeed, in most cases, this response is limited but repetitive injury to ATII cells can lead to exudative inflammation that evokes the recruitment of numerous immune cells and immune dysregulation to promote chronic inflammation [6]. Immune dysregulation is thought to be a driver of interstitial lung diseases. Both innate (macrophages and neutrophils) and adaptive (T cells) cells modulate the fibrogenesis in different ways [7]. As the first barrier in defence against a wide range of inhaled challenges, innate immune cells have important roles in host defence and tissue homeostasis. Macrophages as regulators of pathogenic fibrotic responses are the most-well studied innate immune cells in the onset of PF. Macrophages might induce ECM remodeling via production of TGF-β, angiogenic factors and various cytokines, or attenuate PF through matrix metalloproteinase (MMP)-mediated ECM degradation [8]. Neutrophils produce various proteases, such as serine proteases (neutrophil elastase) and MMPs to degrade ECM but can also active TGF-β, thereby promoting ECM accumulation [1, 8, 9]. In adaptive immune response, some subsets of T cells in the lung might be protective, whereas others accelerate disease progression [2]. Th2 cells produce IL-4, IL-5 and IL-13 to promote the progression of fibrosis, and Th17 cells produce IL-17 can also promote the activation of fibrocytes; on the contrary, Th1 attenuated fibrosis by producing IFN-γ and IL-12 [7]. Tregs may exert both anti- and pro-fibrotic roles, which depending on the balance of Th1/Th2 cells in lung [10, 11]. Although some progress has been made to understand the innate and adaptive immune responses in PF, our grasp of the immune dysfunctions in the pathophysiology of lung diseases is still elusive.

**Fig. 1:**
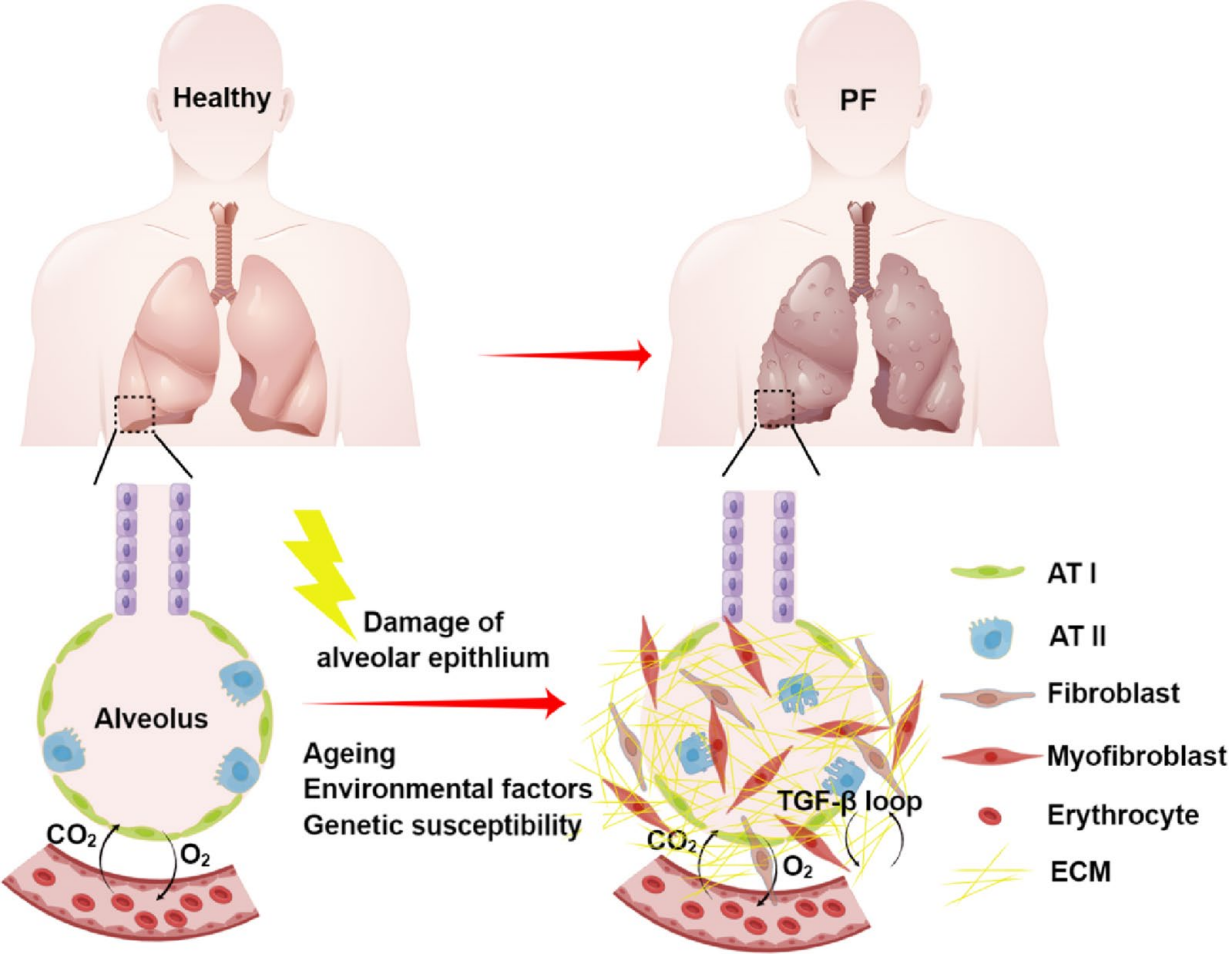
The pathogenetic model of PF. Aging-associated changes, environmental factors and genetic susceptibility result in damage to alveolar epithelial cells. The injured epithelium secretes a plethora of mediators that induce the activation and proliferation of fibroblasts and myofibroblasts, which are resistant to apoptosis and persistently secrete ECM. The ECM also serves as a reservoir of mediators and continuously induces myofibroblast differentiation and ECM secretion in a positive feedback loop, leading to the inexorable progression of PF. By Figdraw (www.figdraw.com)

PF is a complex disorder that results from interactions among risk factors, and the relative contribution of each factor probably differs among individuals. The complexity of PF is also determined by innumerable multidirectional interactions among epithelial cells, mesenchymal cells, and the ECM [2]. On the one hand, the injured alveolar epithelium secretes multiple cytokines and growth factors (e.g., TGF-β) and contributes to the differentiation of fibroblasts into contractile myofibroblasts that can produce ECM. On the other hand, activated fibroblasts/myofibroblasts produce inflammatory mediators, including TGF-β, interleukin (IL)-1 and IL-33, that promote fibrogenesis and recruit immune cells to aggravate chronic inflammation [12]. In addition, the ECM serves as a reservoir of mediators and growth factors that can be released, resulting in a positive feedback loop supporting fibrogenesis through myofibroblast differentiation and ECM secretion. The accumulation of myofibroblasts and ECM in the alveolar space can increase the stiffness and mechanical tension of fibrotic foci, inducing further fibrotic tissue remodeling (Fig. 1) [13].

To date, IPF treatment mainly includes chemical drugs, supplemental oxygen, and organ transplantation. Pirfenidone and nintedanib are the only two drugs approved by the FDA for the treatment of IPF [3, 14]. Pirfenidone is an antifibrotic compound that can target TGF-β, tumor necrosis factor-α (TNF-α), and ILs [14, 15]. Nintedanib is a tyrosine kinase inhibitor that targets vascular endothelial growth factor receptor (VEGFR), FGF receptor (FGFR), and platelet-derived growth factor receptor (PDGFR) [16]. Pirfenidone and nintedanib have shown efficacy in reducing the rate of decline in lung function and slowing disease progression; however, these drugs cannot stop or reverse this disease [17, 18]. In addition, both medications are difficult for patients to tolerate due to adverse effects such as nausea and diarrhea. Currently, lung transplantation is the only feasible curative therapy for patients with advanced PF, but this option is unfortunately limited by the complexity of the surgery and the lack of donors [19]. Therefore, there is a pressing need to develop novel therapeutic agents to prevent or reverse the progression of PF and repair damaged alveolar epithelial cells.

Stem cells have the abilities of self-renewal and multipotent differentiation into different cell types, such as neurons, adipocytes, chondrocytes, osteoblasts, epithelial cells, and muscle cells [20]. Due to their multipotency, low immunogenicity, and paracrine actions, stem cells have been widely used in the treatment of multiple diseases, including diabetes, leukemia, autoimmune diseases, neurodegenerative disorders, and acute and chronic lung injury [21–25]. Stem cells were previously identified as a promising cell type for the treatment of PF. In this review, we will summarize research progress on stem cell-based treatment of PF and analyze concerns regarding stem cell therapy, including ethics, safety, and therapeutic efficacy, to provide a reference for the clinical application of stem cells.

### Lung tissue regeneration capacity of stem cells

Stem cells used for the treatment of PF include endogenous lung stem/progenitor cells, embryonic stem cells (ESCs), induced pluripotent stem cells (iPSCs) and mesenchymal stem cells (MSCs) [25–28]. Next, some central issues with stem cell-based therapy for PF will be summarized.

### Endogenous lung stem/progenitor cells

Early lung stem/progenitor cells are found throughout the pulmonary epithelium and play an important role in the development of the lungs and maintenance of alveolar homeostasis [29]. The lung epithelium contains basal, ciliated, and goblet cells in the proximal airway, as well as neuroendocrine and basal cells in the distal airway. The pseudostratified airway epithelial layer extends to the alveoli and consists of ATII and ATI cells. Basal cells have been considered the lung progenitor cells, and they are characterized by the combined expression of p63, KRT5, and Sox2 [30]. These cells were reported to self-renew and differentiate into ciliated, goblet, club, and epithelial cells in a lung injury model [29–31]. However, the loss of epithelial progenitor cells triggered uncontrolled proliferation of the underlying stroma, leading to the accumulation of fibroblasts and development of obliterative airway lesions [32]. Distal airway stem cells (DASCs), which express the basal cell-restricted transcription factors p63 and KRT5, are also essential for lung regeneration [33, 34]. DASCs have been shown to yield alveoli in vitro following H1N1 influenza infection [35] and to ameliorate bleomycin (BLM)-induced PF in vivo [36]. In addition, perturbations in basal cells and human-iPSC-derived differentiated cells characterize acquired and genetic airway diseases, including mucus metaplasia in asthma, chloride channel dysfunction in cystic fibrosis, and ciliary defects in primary ciliary dyskinesia [37]. These stem cell properties make basal cells a promising candidate for cell-based therapies to restore the airway epithelium.

**Fig. 2:**
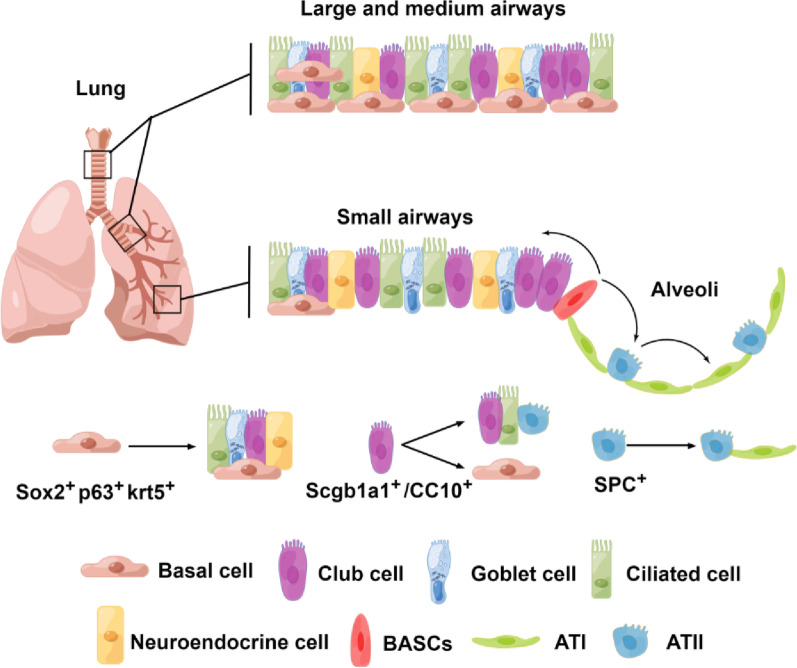
Airway epithelial cells are involved in lung repair and regeneration. The distribution of airway epithelia from the trachea, bronchi and bronchioles to alveoli is shown. Basal cells are progenitor cells of the lungs that are capable of self-renewal and differentiation into club cells, ciliated cells and goblet cells. Club cells can differentiate toward goblet cells, ciliated cells and ATII cells or toward basal cells. BASCs are located at bronchioloalveolar duct junctions (BADJs) and are involved in replenishing club cells and alveolar epithelial cells and promoting the repair and regeneration of lung tissues. ATII cells are progenitor cells of the alveolar epithelium that can self-renew and differentiate into ATI cells. By Figdraw (www.figdraw.com)

Club cells are secretory epithelial cells located in the trachea, proximal bronchus, and distal airway that face the bronchial lumen and have the ability to self-renew and produce ciliated cells. Club cells are cubical in shape and have dense cytoplasmic particles. A molecular feature of the entire club cell population is the expression of secretoglobin family 1A member 1 (Scgb1a1), which responds during epithelial homeostasis and repair after lung injury [38, 39]. Club cell homeostasis is maintained through interactions with ciliated cells [40]. Similar to basal cells, club cells are highly heterogeneous. Classic Scgb1A1^+^ club cells can undergo self-renewal and differentiate into ciliated cells under certain conditions [41]. A subgroup of club cells called variant club cells can resist naphthalene-induced damage and replicate to repair the damaged airway epithelium. Recently, UPK3A was identified as a unique marker of mutant club cells [42]. Studies have shown that variant club cells can help maintain the airway and promote postinjury repair by differentiating into a variety of cell types, including classic club cells and ciliated cells [42, 43]. Interestingly, the results of cell lineage tracing experiment showed that when alveolar epithelial cells are damaged, club cells can differentiate into ATII and ATI cells [44]. ATII cells produce surfactant protein C (SPC), a circulating surfactant protein in alveoli, and are the progenitor cells for ATI cells [45]. Some studies have demonstrated that the transplantation of ATII cells decreased collagen deposition and the severity of BLM-induced PF, even in the context of middle- or late-stage fibrotic lesions [46, 47]. Notably, ATII cells can transiently acquire a transitional state (pre-alveolar type-1 transitional state, PATS) during their differentiation into ATI cells, which is associated with normal epithelial tissue repair and its abnormal persistence in disease conditions in mice [48, 49]. However, it is unclear whether this type of transitional state cells is also existed in primates. Recently, Kadur and colleagues have identified a bipotent progenitor terming alveolar type-0 (AT0) in human lung tissues, another transitional state, that can differentiate into either ATIs or respiratory bronchioles secretory cells (TRB-SCs) during ATIIs’ differentiation [50]. The lineage trajectories of AT0s are distinct from these seen in the mouse lung, which involves a distinct transient state known as PATS. Although, these cells in transitional state during lung tissue regeneration could differ in different species, the transitional progenitors were thought to be involved in many respiratory diseases, including PF and chronic obstructive pulmonary disease (COPD). Previously, we found that transplantation of human ESC/iPSC-derived ATIIs promoted the proliferation of endogenous alveolar progenitors, suggesting that the transplanted human ESC/iPSC-derived ATIIs provided a specific signaling to stimulate the regeneration of BLM-injured lungs in mice [25, 27]. Recently, we showed that human ATII-derived exosome miR-371b-5p promoted ATII proliferation and the re-epithelialization of injured alveoli, indicating miR-371b-5p may serve as a niche signaling to augment ATII proliferation in the injured environment [51]. However, if miR-371b-5p targets the transitional state cells in the specific niche to promote ATII proliferation is unclear, which warrants further studies. In addition, the transitional state cells could serve as a valuable model to select small-molecular drugs that could specifically facilitate the tissue regeneration in the treatment of PF. Zhou's team used genetic lineage tracing to confirm the location of bronchioalveolar duct junctions at the junction of small bronchioles and the alveolar epithelium; in addition, this group reported that cells co-expressing the club cell marker scgb1a1 (or CC10) and the ATII marker SPC, known as bronchioalveolar stem cells (BASCs), can differentiate into ciliated cells after bronchial injury and into ATII cells after alveolar injury, confirming their versatility (Fig. 2) [52]. The discovery of BASCs in vivo as pluripotent stem cells with differentiation ability provides a new direction for cell-based therapy for lung diseases.

Homologous lung stem/progenitor cells also represent a new tool for the personalized treatment of PF as they have shown therapeutic efficacy and safety [36, 53, 54]. The transplantation of homologous lung stem/progenitor cells reduces the risk of immune rejection during clinical treatment, and these cells are specifically differentiated and do not carry the risk of tumorigenicity. However, there is a lack of clinical research on lung stem/progenitor cells, and further studies are warranted. Therefore, more basic and clinical studies are needed to prove the potential of lung stem/progenitor cells in the treatment of PF and other lung diseases.

### Embryonic stem cells

ESCs are pluripotent stem cells isolated from blastocysts that can be induced to generate a variety of specialized cell types [55, 56]. ESCs were shown to differentiate into ATII cells spontaneously in ESC medium, and after enrichment, could serve as a promising transplantable cell type used to treat distal lung injury [57, 58]. Coraux et al*.* showed that murine ESCs could differentiate into alveolar epithelial cells, when the cells were cultured at the air–liquid interface [59], indicating the potential of transplanting human embryonic stem (hES)-ATII cells as an effective strategy to treat the injured epithelium in airway diseases. However, the use of only cell surface markers to identify cell purity is not an accurate strategy. A mixed population of ESC-derived cells will not be suitable for transplantation, and such mixed cultures carry a significant risk of producing teratomas. We have developed a reliable transfection and culture procedure that facilitates the differentiation of hESCs into an essentially pure (> 99%) population of ATII cells that could be used therapeutically to treat distal lung diseases [26]. In addition, we showed that lung injury was abrogated in mice transplanted with hES-ATII cells, as demonstrated by the recovery of body weight and arterial blood oxygen saturation, decreased collagen deposition, and increased survival [27]. All BLM-challenged mice that were treated with hES-ATII cells remained alive and healthy and showed no teratoma formation in the duration of the study. These data indicate that the BLM-injured alveolar epithelium can be functionally and durably repaired by the transplantation of hES-ATII cells. Since it is necessary to destroy embryos to obtain these cells, there are obvious ethical issues; thus, the development and application of ESCs for lung regeneration or to enhance repair have moved more slowly than expected.

### Induced pluripotent stem cells

Similar to ESCs, iPSCs have the capacity for self-renewal and multipotential differentiation into all cell types of the body. In 2006, Takahashi and his colleagues were the first to report that somatic cells could be reprogrammed to an embryonic-like state by inducing the expression of specific pluripotency genes [60]. iPSCs have bright prospects in basic research and the clinical application of cell therapy, as they bypass the ethical concerns associated with the use of hESCs. To date, many investigators have reported that various terminally differentiated human and murine somatic cells can be reprogrammed into iPSCs. Moreover, iPSCs can be induced to differentiate into any cell type responsible for the formation of the human body through gene-editing strategies, including lung epithelial cells and distal progenitor cells [37]. Originally, researchers used basic differentiation medium to induce iPSCs yield basal cells, goblet cells, Clara cells, ciliated cells, and alveolar epithelial cells both in vitro and in vivo [61–65], which may be useful for regenerative medicine; thus, iPSCs are a valuable resource for the treatment of fibrotic lung disease [66].

In defined differentiation medium, human iPSCs (hiPSCs) can differentiate into lung and airway progenitor cells [62] and exhibit ultrastructural characteristics and biological functions similar to those of normal ATII cells in vitro [67]. Soh et al*.* reported that the transplantation of CD166-positive lung epithelial cells derived from hESCs and hiPSCs could abrogate BLM-induced acute lung injury (ALI) by improving pulmonary function and prolonging the survival of mice exposed to BLM. Additionally, cells derived from hESCs and iPSCs express SPC, a specific marker of ATII cells [68]. When the differentiated iPSCs were seeded and cultured in a decellularized mouse lung scaffold, they expressed protein markers, reduced collagen deposition and fibrosis progression, and showed potential for regenerating the three-dimensional (3-D) alveolar lung structure [69]. However, iPSC-derived ATII cells form monolayered epithelial cultures, without the mesenchymal support, have potential to be adapted as a new therapeutic approach for lung injury [64]. Therefore, iPSC-derived lung stem cells provide a platform for disease modeling and future functional regeneration of the distal lung.

The use of lung stem/progenitor cells derived from iPSCs in the repair of BLM-injured lungs has opened the door to new treatments for PF [37, 70]. However, the major obstacles for the clinical application of iPSC-derived cells relate to feasibility and safety, including the long time period required to generate and characterize new cell lines in culture, the retention of the transcriptional memory of the original somatic cell at an epigenetic level, the potential activation of a T lymphocyte-dependent immune reaction, even by autologous iPSCs, and the high risk of tumorigenicity because of the use of oncogenes for reprogramming and the induction of genomic instability [71–75]. Originally, researchers generated iPSCs using genomic integration technologies to remove the subsequent transgene, and subsequent studies demonstrated that iPSCs can be generated in mice and humans without genomic integration [74, 76]. Moreover, in the 10 years since the first report, human iPSCs have become the basis for new cell therapies and drug discovery approaches that have reached clinical application [77]. In one study, correction of the dysfunctional gene in iPSCs generated from cystic fibrosis patients led to differentiation into mature airway epithelial cells [78]. This isogenic iPSC-based model system for cystic fibrosis could be adapted to develop new therapeutic approaches. Various modifications to reprogramming are currently being developed to obtain cell lines without viral vectors and transgene sequences [71]. We reported a novel strategy using a single nonviral, site-specific targeting vector that allowed the efficient generation of patient-specific ATII cells [25]. In the study, hiPSC-ATII cells transplanted into the lungs of BLM-challenged mice with ALI could efficiently restore pulmonary function through re-epithelialization of the injured alveoli, thus preventing lung fibrosis and increasing the survival time without any side effects [25]. Impressively, BLM-injured mice transplanted with hiPSC-ATII cells maintained normal pulmonary function without tumorigenesis for at least 11 months, suggesting that hiPSC-ATII cells are safe and effective in PF therapy [25]. In addition, hiPSCs can be differentiated into alveolar epithelial cells in a rotating bioreactor with an air–liquid interface [79], indicating a new method for the large-scale production of the alveolar epithelium for clinical application. Further genomic, epigenomic, and functional evaluations of cells generated by these new methods are essential to ascertain whether there is a reprogramming procedure that will allow the safe clinical application of iPSCs [80]. However, no report on the clinical application of iPSCs in lung fibrosis has been published; hence, further clinical studies on iPSC-derived lung stem cells for the treatment of IPF are warranted.

### Mesenchymal stem cells

**Fig. 3:**
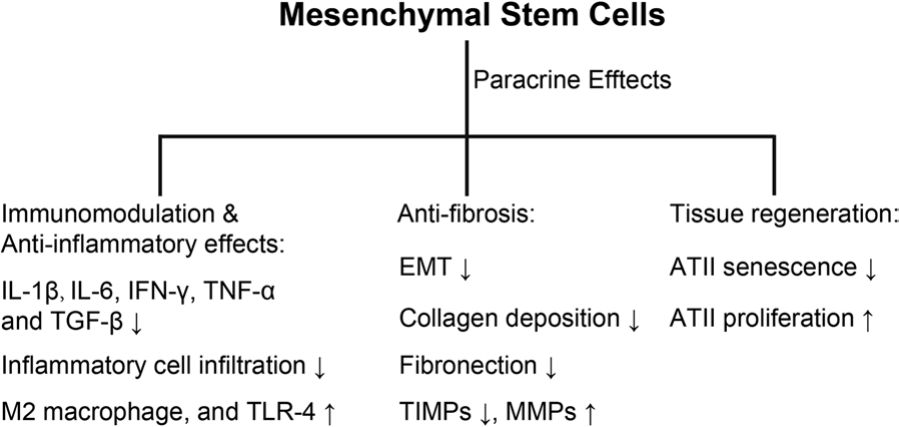
Mechanism of MSC-based therapy for PF. MSCs home to injured lungs, where they exert immunomodulatory and antifibrotic effects via paracrine actions and activate endogenous lung stem cells to promote the regeneration of the injured lungs

MSCs were originally isolated from the bone marrow by Friedenstein [81]. Since then, MSCs have also been obtained from different tissues, such as fat, the umbilical cord, the placenta, amniotic fluid, and cord blood [82–84]. MSCs are multipotent cells with the ability to differentiate into different lineages to repair lung epithelium damaged or destroyed by injury and disease. Initial investigations supported the notion that MSCs, similar to ESCs and iPSCs, engraft and differentiate into alveolar epithelial cells following lung injury [85–87]. However, subsequent studies found that it was difficult for MSCs to engraft and differentiate into alveolar epithelial cells within the injured lungs [88, 89]. Whether MSCs can engraft and differentiate into functional lung cells is controversial and remains to be studied further. Most recent studies have supported the view that MSCs prevent the progression of ALI due to their immunomodulatory and anti-inflammatory functions or promotion of the endogenous tissue regeneration mediated via paracrine actions (Fig. 3) [90–95].

### MSCs used in the treatment of PF

MSCs are widely studied in the field of regenerative medicine, and there are many reports on MSCs for the treatment of PF. Regardless of administration route (systemic administration, intratracheal instillation, or intraperitoneal injection), MSCs can home to injured tissues and target sites of damage [96, 97]. An extensive body of literature on animals and humans supports the therapeutic efficacy of MSCs for the treatment of ALI, PF, and acute pneumonia caused by coronavirus disease 2019 (COVID-19) due to the combination of multipotency, migratory ability, and preservation of immune privilege [98, 99]. In addition, MSCs can improve the microenvironment at the implantation site, which can enhance therapeutic efficacy via paracrine actions [92, 96, 100]. MSC-based therapy is an ideal option for individualized therapy, as these cells can be obtained from various patient sites, such as bone marrow, adipose tissue, and other organs [82]. A summary of preclinical and clinical studies exploring the application of MSCs from different sources in PF treatment is shown in Table 1.

**Table 1:** Preclinical and clinical application of MSCs in PF

Source	Model	Dose, route	Time	Results	References
BMSCs	Mouse, BLM	5 × 10^5^, IV	0, 7 days	Epithelium-like phenotype; inflammation and collagen deposition ↓	[87]
BMSCs	Mouse, BLM	5 × 10^5^, IV	6 h	Lung cell phenotype; inflammation ↓; reparative growth factors ↑	[101]
BMSCs	Mouse, BLM	5 × 10^5^, IV	24 h	MSCs corrected the inappropriate epithelial-mesenchyme relation in IPF	[102]
BMSCs	Mouse, BLM	5 × 10^5^, IV	0 day	ATII cell senescence ↓	[103]
BMSCs	Rat, silica	2 × 10^6^, IV	28 days	E-cadherin and cytokeratin19 ↑; Wnt/β-catenin, Vimentin and α-SMA ↓	[104]
BMSCs	Rat, BLM	1 × 10^6^, IV	14 days	TNF-α, IL-6 and TGF-β ↓	[105]
AMSCs	Mouse, BLM	5 × 10^5^, IV	24 h	Hydroxyproline, α-integrin and TNFα ↓	[106]
AMSCs	Mouse, BLM	5 × 10^5^, IV	24 h	MMP-2, IGF, and AKT ↓	[107]
AMSCs	Mouse, BLM	4 × 10^7^/kg, IV	3, 6, 9 days	Profibrotic and proinflammatory genes ↓	[108]
AMSCs	Mouse, BLM	5 × 10^5^, IV	24 h	miR-199, caveolin-1, and lung fibrosis ↓	[109]
AMSCs	Rat, silica	1 × 10^6^/kg, IV	24 h	TNF-α, IL-1β, IL-6, IL-10, and Caspase-3 ↓; Bcl-2/Bax ratio ↑	[110]
AMSCs	Rat, radiation	5 × 10^6^, IV	2 h, 7 days	EMT, TNF-α, IL-1 and IL-6 ↓; IL-10 and IL-2 ↑	[111]
UCMSCs	Mouse, BLM	1 × 10^6^, IV	24 h	TGF-β, IFN-γ, TNF-α and TIMPs ↓; MMPs ↑	[112]
UCMSCs	Mouse, BLM	5 × 10^5^, IV	0 day	Collagen and fibroblast proliferation ↓; ATII cell proliferation ↑; CXCL9 and CXCL10 ↑	[98]
UCMSCs	Rat, BLM	5 × 10^6^ or 2.5 × 10^7^, IT	21 days	EMT, MMP9, and TLR-4 ↑; released hyaluronan	[95]
UCMSCs	Rat, BLM	2.5 × 10^7^, IT	21 days	Inflammatory cell infiltration and collagen deposition ↓; lung function ↑	[113]
hAMSCs	Rat, WSI	1 × 10^6^, IV	4 h	TNF-α, IL-6 and TGF-β ↓; IL-10, SP-A, SP-C and SP-D ↑	[114]
hAMSCs	Mouse, BLM	1 × 10^6^, IV	15 min	Foxp3 and M2 macrophages ↑; B-cell recruitment, retention, and maturation ↓	[115]
AMSCs	Rat, paraquat	2 × 10^6^, IV	6 h	Collagen, TNF-α, IL-6, TGF-β, and lactic acid ↓	[116]
MenSCs	Mouse, BLM	5 × 10^5^, IV	2 days, 7 days	Collagen, IL-1β, IL-6, IL-10, and TGF-β ↓	[117]
Lung-MSCs	Mouse, BLM	1.5 or 2.5 × 10^5^, IV	0 day	Pulmonary damage and inflammatory cell infiltration ↓	[118]
PMSCs	Mouse, BLM	1 × 10^5^, IV	3 days	Collagen deposition, MyD88/TGF-β and profibrotic cytokines ↓	[119]
PMSCs	Mouse, BLM	4 × 10^6^, IP 1 × 10^6^, IV, IT	15 min	Neutrophil infiltration ↓	[120]
ERC	Mouse, BLM	1 × 10^6^, IV	24 h	Collagen deposition, TGF-β, IL-1β, TNF-α and Bax ↓; IL-10, Bcl-2, HGF and MMP9 ↑	[121]
BMSCs	IPF Patients	20, 100, or 200 × 10^6^, IV		Safe	[122]
BMSCs	IPF Patients	4 × 10^8^, IV		Safe and reduced disease progression	[123]
AMSCs	IPF Patients	5 × 10^5^/kg, IT		Safe	[124]
UCMSCs	IPF Patients	1 or 2 × 10^6^/kg, IV		Feasible and safe	[125]

## Summary

For lung stem/progenitor cells, ESCs and iPSCs, many factors, including the number of obtained cells, ethical issues, safety and the lengthy time required for cell preparation, limit their clinical application in the treatment of PF. MSCs are currently the most commonly used stem cells in clinical trials because of their low immunogenicity and tumorigenicity and the lack of potential ethical problems [126]. However, some problems should be considered in clinical research on MSCs.

### Considerations in clinical trials of MSCs

Previously, we found that the intratracheal administration of ATII cells derived from ESCs and iPSCs effectively abrogated BLM-induced ALI [25, 27]. However, the systemic administration, but not the intratracheal instillation, of human umbilical cord (UC)-MSCs blocked the progression of BLM-induced lung injury in mice, and repeated intravenous administration of a low dose of UCMSCs reversed fibrotic scarring in lung tissues. These findings suggest that in the clinical application of MSCs and other types of stem cell therapy, the appropriate delivery route, dose, and administration frequency are key considerations.

The delivery route is the main factor that affects the transport of cells to target organs [127]. In preclinical and clinical studies of MSC-based PF treatment, the delivery routes have mainly included intravenous injection, intratracheal instillation, and intraperitoneal injection [95, 108, 120]. Intravenous injection has been the main delivery route in most studies, as it is minimally invasive, simple to perform, and the most common method of MSC administration in the treatment of various lung diseases [128]. In addition, intravenously administered MSCs can receive signals released by damaged tissues, thus inducing cell homing to the injured areas [129]. Therefore, systemic intravenous administration may be a suitable delivery route for MSC transplantation. Compared with intravenous injection, intratracheal delivery ensures that most of the transplanted stem cells migrate to the injured tissues, and some studies have used intratracheal instillation to transplant MSCs into the lungs of animals with PF [95]. Local delivery and transportation are widely used for ESC- or iPSC-derived lung progenitor cells [25, 64] because intratracheal instillation can directly deliver cells to the damaged tissues, reducing the side effects of cells on other organs, and the cells can differentiate into functional cells to repair the injured lungs. In addition, the intraperitoneal injection of MSCs has been reported to have a certain preventive effect on ALI and PF [120, 130]. In clinical practice, pulmonary fibrotic foci are scattered in lung tissue rather than concentrated at a certain point, which may be inconvenient for intratracheal transplantation. In addition to traditional systemic intravenous injection and intratracheal instillation, nebulization can uniformly distribute drugs in injured lung tissues [131, 132]. However, in stem cell-based therapy for PF, the use of nebulization is limited to the delivery of stem cell-derived extracellular vesicles (EVs)/exosomes. Although some studies explored the feasibility of nebulization-based delivery of stem cells into lungs, which might not apply here in PF therapy due to a significant loss of cell viability as reported [133, 134]. In general, the transplantation route for MSC-based PF treatment needs to be explored further.

In addition to the appropriate delivery route, the number of cells that play a therapeutic role in damaged tissue should also be considered. Different doses of MSCs have been used in various preclinical and clinical studies. Some preclinical studies have shown that the effective dose of MSCs in rodents is ranged from 0.1 × 10^7^ to 5 × 10^7^ cells/kg [95, 110, 112]. By analyzing clinical trials involving MSCs, Kabat et al*.* showed a narrower minimum effective dose (MED) range of 1–1.5 × 10^8^ MSCs/patient among trials that reported positive results [135]. Some recent clinical trials showed that the intravenous administration of 1–4 × 10^6^ MSCs/kg weekly is feasible and safe in patients with organ transplantation or COVID-19 [136–138]. The clinical therapeutic effects are lost at low doses of MSCs, while a high dose of transplanted MSCs may increase the risks of pulmonary embolism and inflammation, thus increasing the phagocytosis of macrophages and reducing the therapeutic effect [95, 139–141]. Therefore, it is necessary to comprehensively evaluate the MED before clinical application.

BLM-induced ALI and fibrosis in mice is a well-established model known to mimic the onset of human lung fibrosis that proceeds through the following three stages: the inflammatory stage, the fibroblast proliferation stage, and the interstitial remodeling stage [142, 143]. Two recent preclinical studies have used BLM to establish animal models of ALI and evaluate the therapeutic effect of stem cells [27, 87]. Some studies showed that in the early stage, stem cell transplantation effectively abrogated BLM-induced ALI by reducing inflammatory cell infiltration and collagen deposition and promoting the repair of injured lungs [98, 102, 109, 115]. However, most studies performed to date have focused on the capacity of stem cells to rescue or prevent the occurrence of PF. Unfortunately, in clinical practice, most patients with interstitial lung disease already have varying degrees of lung fibrosis. Therefore, the repair of damaged lung tissues and the ability to reverse the loss of pulmonary function are critical goals in the treatment of PF. Gad et al*.* showed that on day 14 after BLM instillation, the transplantation of MSCs inhibited the expression of TGF-β/Smad3 and reduced the deposition of collagen in BLM-injured lungs in rats [105]. Chu et al*.* reported that the endotracheal administration of a single dose (2.5 × 10^7^) of UCMSCs reduced BLM-induced fibrosis by inducing the synthesis of MMP-9 and a decrease in collagen deposition in a rat model of late-stage IPF [95, 113]. Furthermore, the therapeutic effect of MSCs in an animal model of silica-induced fibrosis was promising [104]. The results of this study suggested that a single administration of stem cells could prevent the progression of ALI and attenuate the development of advanced PF induced by BLM; however, it was unable to restore the damaged lungs to normal conditions. In fact, MSCs were entrapped in the lungs and were short-lived after transplantation [141, 144]. Within 24 h after administration, MSCs activated hypoxia signaling pathways and caspase-3-mediated apoptosis, followed by the local recruitment of immune cells to the transplantation site and the engulfment of apoptotic MSCs by macrophages [141]. Therefore, it is necessary to consider the repeated transplantation of stem cells to ensure long-term effects. Interestingly, the repeated administration of MSCs had comparable antifibrotic effects to the continuous administration of pirfenidone. Similar results were observed in a rat model of radiation-induced lung fibrosis [108, 111, 117]. Repeated infusions of MSCs for the treatment of IPF have not been evaluated in clinical trials, although this administration strategy has been applied to other diseases [136, 137, 145]. Therefore, multiple rounds of MSC transplantation may be a promising approach for the treatment for PF. However, most clinical trials of MSC-based therapies for PF have not yet been completed and are still in phases I and II. More clinical trials need to be performed to realize the clinical application of MSCs for the treatment of PF.

### MSC-derived extracellular vesicles in the treatment of PF

A growing body of literature suggests that stem cell conditioned medium containing EVs can replicate the therapeutic effects of stem cells. Exosomes, a key type of EV, are small cell-derived EVs with a diameter of 30–100 nm that are enriched with specific marker proteins, including calcium-dependent phospholipid-binding proteins, heat-shock proteins (Hsp60, Hsp70, and Hsp90), transmembrane proteins (CD63, CD81, and CD9), and others [146]. Exosomes act as carriers of various functional proteins, mRNAs, and microRNAs and serve as key mediators of intercellular communication [51]. Compared with stem cells, exosomes offer more advantages in tissue regeneration and have lower risks of immune rejection, tumorigenicity, and pulmonary embolism, which can be caused by stem cells [147]. Therefore, exosomes show similar or even better therapeutic effects than stem cells in PF. We have summarized studies on MSC-derived exosomes in the treatment of PF in Table 2. In addition, EVs from lung stem/progenitor cells or other cells are also shown.

**Table 2:** Studies of stem cell-derived EVs in PF

Source of EVs	Model	Dose, route	Time	Results	References
WJMSCs	Mouse, HYRX	IV	4 days	Fibrosis ↓; modulated M1/M2	[92]
hAECs	Mouse, BLM	10 μg, IT	1 day	Anti‐inflammatory, antifibrotic, and pro‐regenerative	[148]
hAECs	Mouse, BLM	5 or 25 μg, IT	3 weeks	Improved PF	[149]
BMSCs	Mouse, BLM	8.6 × 10^8^, IV	7, 21 days	Immunoregulatory and anti-inflammatory	[100]
LSCs	Mouse, BLM or Silica	Nebulization	BLM 10 days, silica 28 days	Collagen and myofibroblast proliferation ↓	[131]
PMSCs	Mouse, radiation	100 μg, IV	0, 3, 5, 7 days	Inflammation, fibrosis, and DNA damage ↓	[150]
Macrophages	A549, MRC5		24 h	Antifibrotic via miR-142-3p	[151]
WJMSCs	Mouse, HYRX	100 μL, IV	18–39 days	Improved PF	[152]
UCMSCs	Mouse, silica	200 μg, IV	Every 4 days	Collagen I and fibronectin ↓	[153]
UCMSCs	Mouse, BLM	20 μg, IV	7, 21 days	Fibrosis ↓; AEC proliferation ↑	[154]
HBECs	Mouse, BLM	2 × 10^9^, IT	7, 14 days	β-Catenin and cell senescence ↓	[155]
AMSCs	Mouse, *P.* aeruginosa; human volunteers	Mouse 1 mL; human 2 to 16 × 10^8^, Nebulization	2 h, mouse	Survival rate of mice with *P. aeruginosa*-induced lung injury ↑; well tolerated by volunteers	[132]
BMSCs	Mouse, silica	10 μg, IV	14 weeks	Collagen and inflammation ↓	[156]
AMSCs	Mouse, silica	50 μL, IT	15 days	Collagen and inflammation ↓; IL-1β, IL-6, TGF-β, and TNF-α ↓	[157]

## Conclusions

In this review, we summarized fibrogenesis in PF, highlighting the roles of stem cells derived from different sources in the repair of fibrotic lung tissues. Then, we analyzed some key concerns that should be considered in the clinical treatment of PF with different types of stem cells, such as the administration route, dose and frequency. In addition, the superiority of and current problems with stem cell-derived exosomes for the treatment of PF were also discussed. To conclude, we speculated that stem cell-based therapy has great promise for PF.

Despite this compilation of research, much is still unknown about the mechanisms of stem cells in the treatment of fibrotic disease. While it is thought that inflammation and fibrosis are perpetuated by lung injury, how stem cells fit into the picture, exert anti-inflammatory activity, and participate in the reversal of fibrosis remain unclear. Lung stem/progenitor cells and ESCs or iPSCs repair fibrotic lung tissues by differentiating into functional alveolar epithelial cells [25–27, 54]. In contrast to lung stem/progenitor cells or pluripotent stem cells, MSCs do not differentiate into alveolar epithelial cells; rather, they improve host immunity, degrade ECM, and promote the regeneration of endogenous distal pulmonary stem cells via paracrine actions [95, 100]. In reported experiments, animal models of lung fibrosis induced by the intratracheal administration of BLM, silica, or radiation or by hypoxia were used to highlight the roles of stem cells in the prevention and treatment of this disease [101, 131, 150, 152]. Although these animal models are useful for studying fibrogenesis in PF and we hope the mechanisms are shared between rodents and humans, the occurrence of human PF is very complicated, and it should be acknowledged that none of these animal models fully recapitulate the natural course of human lung fibrosis. Additionally, in most previous preclinical trials, stem cells were administered once or at the early stage of induced lung inflammation, which limited the focus to the capacity of stem cells to rescue acute inflammation or prevent the development of lung fibrosis [95, 98]. In contrast, most patients with PF who present at a clinic already have middle- or end-stage PF. Therefore, selecting an appropriate animal model is imperative for evaluating the therapeutic effect of stem cells.

MSCs are the most commonly studied stem cells in clinical trials. Intravenous injection is considered the main delivery route for stem cells due to the advantages of being minimally invasive and simple to perform; thus, intravenous injection is the most commonly used method to administer UCMSCs in various pulmonary diseases [128]. The safety and therapeutic efficacy of MSCs have been reported, but only in phase I/II studies, and more extensive clinical evaluation is needed. Interestingly, the half-life of intravenously administered MSCs is short, as the apoptotic pathway is immediately initiated, and the cells are cleared by macrophages within 7 days. Thus, multiple infusions of effective doses of MSCs may offer hope for patients with PF.

Genetic risk factors, such as MUC5, telomerase, and surface-active protein, have been confirmed to be related to the onset of PF [3, 158]. Therefore, the combination of stem cell therapy and gene therapy may offer new hope for the treatment of PF. It is necessary to evaluate the genetic information of donors and then genetically modify stem cells to enhance their targeting and therapeutic effect. Some recent preclinical studies reported that the regulation of gene expression related to the development of lung fibrosis plays a positive role in the repair of damaged lung tissues [159–161]. Moreover, the administration of genetically engineered stem cells effectively protected mice from pulmonary infection [162]. Although studies have shown that MSCs can improve the lung function of patients with PF, they cannot eliminate fibroblasts, degrade unwanted ECM, or regenerate the alveolar epithelium. In contrast, stem cell-derived exosomes show potential in the treatment of advanced lung fibrosis. The safety and effectiveness of exosomes in the treatment of IPF have also been confirmed [92, 100, 132]. However, most of the exosomes used in these studies were directly obtained from normal MSC medium and thus lacked tissue specificity and had a poor therapeutic effect. The finding may be explained by the fact that stem cells secrete specific EVs that target the injured tissue in the local lung microenvironment. We previously showed that exosomal miRNA-371b-5p promoted the proliferation of distal lung stem cells and repaired injured lungs, while exosomes obtained directly from the medium lacked the ability to repair damaged lung tissue [51]. It is not easy to obtain specific stem cell-derived exosomes from the complex lung environment, but this should be the direction of stem cell therapy.

## Data Availability

All data are available on request.

## Abbreviations

PF: Pulmonary fibrosis
IPF: Idiopathic pulmonary fibrosis
EVs: Extracellular vesicles
Exos: Exosomes
ECM: Extracellular matrix
ALI: Acute lung injury
AT0: Alveolar type-0
ATI: Alveolar type I
ATII: Alveolar type II
TGF-β: Transforming growth factor-β
FGF: Fibrogenic growth factors
TNF-α: Tumor necrosis factor-α
VEGFR: Vascular endothelial growth factor receptor
FGFR: Fibroblast growth factor receptor
PDGFR: Platelet-derived growth factor receptor
ESCs: Embryonic stem cells
iPSCs: Induced pluripotent stem cells
MSCs: Mesenchymal stem cells
DASCs: Distal airway stem cells
BADJs: Bronchioloalveolar duct junctions
BLM: Bleomycin
Scgb1a1: Secretoglobin family 1A member 1
SPC: Surfactant protein C
CC10: Secretoglobin1a1
BASCs: Bronchioalveolar stem cells
COVID-19: Coronavirus disease 2019
BMSCs: Bone marrow mesenchymal stem cells
AMSCs: Adipose-derived mesenchymal stem cells
UCMSCs: Umbilical cord mesenchymal stem cells
AMSCs: Amnion-derived mesenchymal stem cells
MenSCs: Menstrual blood-derived stem cells
PMSCs: Placental mesenchymal stem cells
ERC: Endometrial regenerative cells
WJMSCs: Wharton's jelly-derived mesenchymal stem cells
hAECs: Human amnion epithelial cell
LSCs: Lung spheroid cell
HBECs: Human bronchial epithelial cells
IV: Intravenous
IT: Intratracheal
MED: Minimum effective dose
MMP: Matrix metalloproteinase
PATS: Pre-alveolar type-1 transitional state
TRB-SCs: Respiratory bronchioles secretory cells
COPD: Chronic obstructive pulmonary disease
